# Clinical Presentation, Management, and Outcomes of Access-Related Radial Artery Pseudoaneurysms: A Single-Center, Retrospective Cohort Study

**DOI:** 10.1016/j.jscai.2025.104065

**Published:** 2025-12-09

**Authors:** Michael Vlach, Aqsa Choudhry, Rhythm Vasudeva, Mohinder Vindhyal, Prasad Gunasekaran, Gaurav Parmar, Georges Haj, J.D. Serfas, Mark Wiley, Peter Tadros, Eric Hockstad, Anthony Spaedy, Seth Decamp, Kamal Gupta

**Affiliations:** a Department of Internal Medicine, The University of Kansas Medical Center, Kansas City, Kansas; b Department of Cardiovascular Medicine, The University of Kansas Medical Center, Kansas City, Kansas; c Section of Vascular Medicine, Massachusetts General Hospital, Harvard Medical School, Boston, Massachusetts; d Division of Vascular Surgery, Department of Surgery, The University of Kansas Medical Center, Kansas City, Kansas

**Keywords:** access site complications, coronary angiography, radial artery pseudoaneurysm

## Abstract

**Background:**

Radial artery pseudoaneurysm (RAP) is a rare complication of procedural radial access. There is a paucity of data regarding clinical presentation, characteristics, and management.

**Methods:**

We identified patients with RAP by querying our center’s electronic medical records. A manual chart review was performed, and a descriptive analysis was conducted.

**Results:**

We identified 35 patients with RAP (mean age, 68.3 years; 62.9% women). Of these, 71.4 % of pseudoaneurysms were caused by arterial access for coronary procedures. Sixty percent were on anticoagulation. The most common symptoms were swelling (88.6%) and pain (45.7%). The median time from procedure to diagnosis was 13 days (IQR, 1-33.5 days), and the median time from procedure to symptom onset was 1 day (IQR, 0-8.5 days). Initial treatment modality was mechanical compression in 19 patients (54.3%). Of those who underwent compression, the RAP thrombosed in 9 patients (47.4%) and failed in 10 patients (52.6%) who then underwent successful surgical repair. Those with RAP resolution with compression had a shorter time to ultrasound diagnosis (1 vs 6.5 days) and smaller size (1.6 vs 2.4 cm). Surgery was successful in all but 1 patient.

**Conclusion:**

Study findings show that RAPs often present several days after the index procedure, although symptoms occur much earlier. The RAPs occur disproportionately in women and those on anticoagulation. Compression is successful in only half the patients. Earlier presentation and smaller size predict success. Surgery is almost universally successful, and thrombin injection is rarely used. Our results suggest a need for prospective studies to assess strategies for earlier identification of RAP.

## Introduction

Over the last 2 decades, there has been a steady increase in the utilization of radial artery access (RAA) for cardiac catheterization procedures. This is now the predominant access site for coronary angiography and interventions in the United States.^1^^,^^2^ The RAA is associated with significantly lower rates of local vascular complications, such as bleeding, pseudoaneurysm, hematoma, and reduced mortality, compared with the femoral approach, with similar procedural outcomes.^3^, ^4^, ^5^, ^6^

Available literature suggests that pseudoaneurysm formation after radial access is relatively uncommon. A retrospective study of almost 15,000 patients undergoing percutaneous coronary procedures found the incidence of radial artery pseudoaneurysm (RAP) to be about 0.08%, which is significantly lower than the incidence with femoral access of about 1.4%.^7^^,^^8^ Besides cardiovascular procedures, bedside radial arterial lines may also cause radial artery injury and may result in pseudoaneurysm formation.

The predictors and management strategies for pseudoaneurysms from femoral artery access have been extensively researched.^9^, ^10^, ^11^ However, apart from case reports and relatively small case series, there is a remarkable paucity of data on the clinical presentation, management strategies, and outcomes of RAA-related pseudoaneurysms in routine clinical practice.^12^^,^^13^ Furthermore, even less is known about the predictors of the success of various treatment strategies of RAP. Given the paucity of data, we conducted this single-center retrospective study to investigate the clinical presentation, patient characteristics, management approaches, and outcomes in patients who develop RAP after percutaneous arterial access.

## Materials and methods

This retrospective, longitudinal study was conducted at the University of Kansas Medical Center. This study was approved by the institutional review board (STUDY00160953). We identified patients with documentation of radial artery pseudoaneurysm by querying our center’s electronic medical records with the Illuminate InSightnatural language processing software with the query “(Radial Pseudoaneurysm OR Radial Aneurysm) AND (Document type [Cardiology]).” Each query result was then manually reviewed by a study team member to confirm that the patient did have a RAP confirmed by duplex sonography. Two study team members then independently conducted a manual chart review to collect the study data points. Any discrepancies were resolved by discussion among the investigators. Demographic characteristics, clinical characteristics, comorbidities, procedural details, and management details were collected in a RedCap database and stored in a password-protected and institution-approved share drive. Continuous data were described as mean, median, or interquartile range, depending on the distribution. Categorical data were described as frequencies and percentages.

Comparisons between categorical variables were made using Fisher exact tests. Continuous variables were compared using the *t* test for most variables with reported means and the Kruskal-Wallis test for time-related variables, as they were determined to have non-normal distributions. Analysis was performed using “R” statistical software (R Studio 2021.09.0 Build 351), which is an open-source, free software environment for statistical computing and graphics. Due to the small sample size, we deemed that the tests of statistical significance were not meaningful and thus did not report them in the main manuscript. They are instead reported in Supplemental Tables S1 and S2.

A descriptive analysis was then conducted. Subgroup comparisons were performed between those treated with the initial compression strategy versus other modalities and between those with successful compression versus those without successful compression to examine factors playing a role in successful compression therapy.

## Results

### Patient characteristics

We identified 35 patients with confirmed RAP in our electronic medical records (January 11, 2011, to June 27, 2024) who were determined to be arterial access-related. Demographic characteristics and baseline comorbidities are detailed in Table 1. The mean age was 68.3 years; the mean body mass index (BMI) was 29.2 kg/m^2^. In total, 62.9% (22) of the patients were women, and most were White (94.3%). Common comorbidities were hypertension (68.6%), congestive heart failure (42.9%), hyperlipidemia (45.7%), prior coronary artery disease (48.6%), and a history of smoking (42.9%).

**Table 1:** Demographics and clinical characteristics of patients diagnosed with iatrogenic pseudoaneurysm.

Characteristic	N = 35
Age, y	68.3 ± 15.6
Sex	
Male	13 (37.1%)
Female	22 (62.9%)
Race	
Black or African American	2 (5.7%)
White	33 (94.3%)
Weight, kg	81.9 ± 28.5
Height, cm	166.7 ± 11.9
Body mass index, kg/m^2^	29.2 ± 9.0
Hypertension	24 (68.6%)
Diabetes	7 (20.0%)
Peripheral arterial disease	4 (11.4%)
Congestive heart failure	15 (42.9%)
Hyperlipidemia	16 (45.7%)
Coronary artery disease	17 (48.6%)
Tobacco use	15 (42.9%)

Values are mean ± SD or n (%).

Of the total study population of 35 patients, 18 (51.4%) were on a single antiplatelet agent, 10 (28.6%) were on dual antiplatelet therapy, and 21 (60%) patients were on therapeutic anticoagulation.

### Radial pseudoaneurysm details

Table 2 provides the details of RAP-related clinical presentation, RAP anatomy, management, and outcomes. The median time from causative procedure to ultrasound diagnosis was 13 days (IQR, 1-33.5 days). The median time from causative procedure to symptom onset was 1 day (IQR, 0-8.5 days). The most common presenting symptoms were swelling (88.6%) and pain (45.7%). No patient had evidence of distal embolization to the hand or symptoms of hand ischemia. The Central Illustration describes the causative procedures for the RAP in the study population. A majority of RAPs (71.4%) were caused by arterial access for coronary procedures. Others were related to arterial blood gas measurements (8.6%), arterial lines (14.3%), or other vascular procedures (1 aortic angiogram and 1 subclavian artery intervention). Of the patients who had access to coronary procedures, ultrasound-guided RAA was used in 7 (28%). Percutaneous coronary intervention was performed in 5 patients.

**Table 2:** Characteristics pertaining to pseudoaneurysm diagnosis.

Characteristic	N = 35
Time to ultrasound diagnosis^a^, d	13 (1-33.5)
Time to symptom onset^b^, d	1 (0-8.5)
Ultrasound diagnosis within 7 d from causative procedure	14 (40%)
Symptom	
Pain	16 (45.7%)
Swelling	31 (88.6%)
Numbness/tingling	2 (5.7%)
Bruising/discoloration	2 (5.7%)
Causative procedure	
Heart catheterization	25 (71.4%)
Arterial blood gas	3 (8.6%)
Arterial line	5 (14.3%)
Other	2 (5.7%)
PCI (for patients undergoing heart catheterization)	5 (20%)
Ultrasound-guided radial access	7 (28%)
Initial use of TR band post heart catheterization	24 (96%)
Initial size—max diameter, cm	2.21 ± 1.7
Presence of hematoma	3 (8.6%)
Initial treatment with compression	19 (54.3%)
Time to compression from symptom onset, d	
Mean	2.9 ± 7.2
Median	0 (0-0.50)
Time to compression from causative procedure, d	
Mean	8.1 (15.4)
Median	1 (0-6.5)
Compression success	9 (47.4%)
Treatment other than compression	21 (60.0%)
Type of treatment (other than compression)	
Thrombin injection	1 (4.8%)
Surgical repair	20 (95.2%)
Time to surgery from symptom onset, d	16.0 (5.2-65.0)
Time to surgery from causative procedure, d	30.5 (13-68.8)
Surgical success	19 (95%)
Exposure to anticoagulation	21 (60.0%)

Values are median (IQR), mean ± SD, or n (%).

PCI, percutaneous coronary intervention; TR, Terumo Radial.

a Time from causative procedure to ultrasound diagnosis.

b Time from causative procedure to symptom onset.

**Central Illustration:**
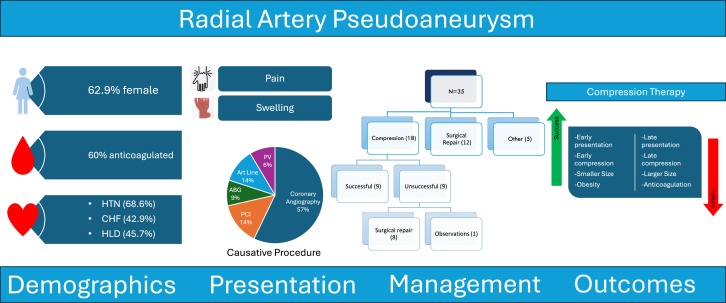
**Clinical characteristics, management, and outcomes of access-related radial artery pseudoaneurysm.** Summary of key clinical findings. ABG, arterial blood gas; CHF, congestive heart failure; HLD, hyperlipidemia; HTN, hypertension; PCI, percutaneous coronary intervention; PV, peripheral vascular.

The various management strategies for the study population are shown in the Central Illustration. Local compression was the initial treatment modality in 19 patients (54.3%). The mean time from symptom onset to compression was 2.9 days. The mean time to compression from the causative procedure was 8.1 days. Compression was successful in 9 patients (47.4%). Of the 10 patients who failed compression, 9 subsequently underwent surgical repair, and one was clinically observed without further intervention. In the 16 patients (45.7% of the 35 total patients) not treated initially with compression, 11 patients were treated with surgical repair, 4 patients were managed with observation alone, and 1 patient was treated with thrombin injection (successfully).

Overall, 20 patients underwent surgical repair as an initial treatment strategy or after failed compression therapy. The median time from symptom onset to surgical repair was 16 days. Nineteen patients had successful repairs defined as ultrasonographic or clinical resolution at follow-up. One patient had persistence of a pseudoaneurysm after repair at follow-up. This was managed with clinical observation, and the patient had resolution of symptoms on follow-up, but further imaging was not done. One patient had wound dehiscence after repair, requiring repeat surgery.

In order to understand the differences between patients where physicians chose compression as the initial treatment modality versus where they did not, we performed a comparison of the 2 groups (Table 3). Patients who had compression as initial treatment presented earlier (median time to ultrasound diagnosis 1 vs 33.5 days), and a greater percentage had cardiac catheterization as the causative procedure (15 patients, 78.9% vs 10 patients, 62.5%) than in the noncompression group. The mean diameter of the pseudoaneurysm was 2.0 cm in the initial compression group vs 2.4 cm in the noncompression group. Initial compression was deemed successful in 9 patients (47.4%) and unsuccessful in 10 patients (52.6%). Of these, 9 patients underwent surgical correction, and 1 patient was clinically observed.

**Table 3:** Patient characteristics and pseudoaneurysm details for patients who underwent compression vs those who did not for initial management of radial pseudoaneurysm.

	Initial compression (n = 19)	No initial compression (n = 16)
Age, y	65.6 ± 18.7	71.6 ± 10.5
Sex		
Male	7 (36.8%)	6 (37.5%)
Female	12 (63.2%)	10 (62.5%)
Race		
Black or African American	1 (5.3%)	1 (6.3%)
White	18 (94.7%)	15 (93.8%)
Weight, kg	83.6 ± 32.0)	80.0 ± 24.6
Height, cm	167.1 ± 12.1)	166.3 ± 12.0
Body mass index, kg/m^2^	29.7 ± 9.9	28.7 ± 8.1
Comorbidities		
Hypertension	15 (78.9%)	9 (56.3%)
Diabetes	3 (15.8%)	4 (25.0%)
Peripheral arterial disease	4 (21.1%)	0 (0%)
Congestive heart failure	9 (47.4%)	6 (37.5%)
Hyperlipidemia	9 (47.4%)	7 (43.8%)
Coronary artery disease	10 (52.6%)	7 (43.8%)
Tobacco use	9 (47.4%)	6 (37.5%)
Pseudoaneurysm details		
Time to ultrasound diagnosis, d	1.0 (0-11.5)	33.5 (21.8-62.5)
Symptom		
Pain	10 (52.6%)	6 (37.5%)
Swelling	16 (84.2%)	15 (93.8%)
Numbness	0 (0%)	2 (12.5%)
Bruising/discoloration	1 (5.3%)	1 (6.2%)
Causative procedure		
Heart catheterization	15 (78.9%)	10 (62.5%)
Arterial blood gas	1 (5.3%)	2 (12.5%)
Arterial line	2 (10.5%)	2 (12.5%)
Other	1 (5.3%)	2 (12.5%)
Undergone PCI—among patients with heart catheterization	1 (6.7%)	4 (40%)
Ultrasound-guided arterial access	4 (26.7%)	3 (30%)
Initial size—max diameter, cm	2.0 ± 1.5	2.4 ± 2.0
Presence of hematoma	3 (15.8%)	0 (0.0%)
Compression success	9 (47.4%)	—
Treatment with intervention	9 (47.4%)	12 (75%)
Intervention type		
Thrombin injection	0 (0%)	1 (8.3%)
Surgical repair	9 (100%)	11 (91.7%)
NA	10	4
Time to surgery from symptom onset, d	6 (3.0-14.0)	65 (16.0-92.0)
Time to surgery from causative procedure, d	13 (4.0-19.0)	67 (41.0-105.0)
Surgery success	9 (100%)	10 (90.9%)
Exposure to anticoagulation	11 (57.9%)	10 (62.5%)

Values are mean ± SD, n (%), or median (IQR).

NA, not applicable; PCI, percutaneous coronary intervention.

**Figure 1:**
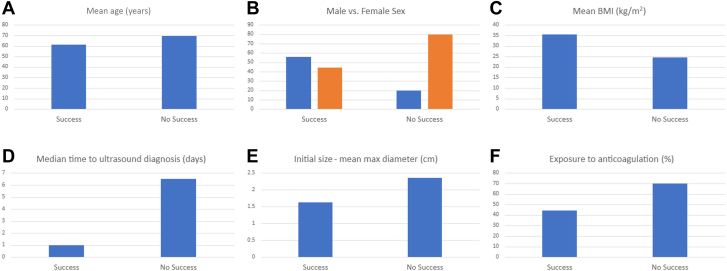
**Comparison of various variables in successful versus unsuccessful compression therapy**. (A) Age. (B) Sex. (C) Body mass index (BMI). (D) Time to diagnosis. (E) Initial size expressed as the mean max diameter. (F) Exposure to anticoagulation.

In order to understand the factors determining the success of initial compression therapy, we further compared groups where initial compression was successful versus where it was unsuccessful (Table 4, Figure 1). The group that had success with compression treatment was younger (mean age, 61.3 vs 69.4 years), had higher BMI (35.4 vs 24.5 kg/m^2^), presented earlier after the causative procedure (median time to ultrasound diagnosis, 1 vs 6.5 days), and had smaller initial maximum RAP diameter (1.6 cm vs 2.4 cm). The mean time from symptoms to compression was shorter in those with successful compression (0.8 vs 4.8 days). The time from causative procedure to compression was also shorter (7.1 vs 9 days). In the successful compression group, 44.4% of patients were on anticoagulation, whereas 70% in the unsuccessful compression group were anticoagulated.

**Table 4:** Patient characteristics and pseudoaneurysm details for patients in whom compression was successful vs not when used for initial management of radial pseudoaneurysm.

	Success (n = 9)	No success (n = 10)
Age, y	61.3 ± 17.0	69.4 ± 20.3
Sex		
Male	5 (55.6%)	2 (20.0%)
Female	4 (44.4%)	8 (80.0%)
Race		
Black or African American	0 (0%)	1 (10.0%)
White	9 (100%)	9 (90.0%)
Weight, kg	101.8 ± 34.4	67.2 ± 19.2
Height, cm	169.0 ± 9.8	165.3 ± 14.1
Body mass index, kg/m^2^	35.4 ± 10.8	24.5 ± 5.4
Comorbidities		
Hypertension	7 (77.8%)	8 (80.0%)
Diabetes	2 (22.2%)	1 (10.0%)
Peripheral arterial disease	1 (11.1%)	3 (30.0%)
Congestive heart failure	5 (55.6%)	4 (40.0%)
Hyperlipidemia	4 (44.4%)	5 (50.0%)
Coronary artery disease	7 (77.8%)	3 (30.0%)
Tobacco use	6 (66.7%)	3 (30.0%)
Pseudoaneurysm details		
Time to ultrasound diagnosis, d	1.00 (0-1.0)	6.50 (1.3-16.8)
Ultrasound diagnosis within 7 d from causative procedure	8 (88.9%)	5 (50%)
Symptom		
Pain	6 (66.7%)	4 (40.0%)
Swelling	9 (100%)	7 (70.0%)
Bruising/discoloration	0 (0%)	1 (10.0%)
Causative procedure		
Heart catheterization	8 (88.9%)	7 (70.0%)
Arterial blood gas	0 (0%)	1 (10.0%)
Arterial line	1 (11.1%)	1 (10.0%)
Other	0 (0%)	1 (10.0%)
Undergone PCI—among patients with heart catheterization	1 (12.5%)	0 (0%)
Ultrasound-guided arterial access	2 (25.0%)	2 (28.6%)
TR band placed (at any time)	9 (100%)	9 (90.0%)
Initial use of TR band (post-LHC)	8 (88.9%)	8 (80.0%)
Initial size—max diameter, cm	1.6 ± 1.3	2.4 ± 1.6
Presence of hematoma	2 (22.2%)	1 (10.0%)
Exposure to anticoagulation	4 (44.4%)	7 (70.0%)
Time to compression from symptom onset, d		
Mean	0.8 ± 2.0	4.8 ± 9.6
Median	0 (0-0)	0 (0-2.3)
Time to compression from causative procedure, d		
Mean	7.1 ± 19.5	9 ± 11.5
Median	1 (0-1)	2.5 (0.3-16.0)
Treatment with intervention	—	9 (90.0%)
Intervention type		
Surgical repair	—	9 (100%)
Time to surgery from symptom onset, d		
Mean	—	9.7 ± 10.0
Median	—	6.0 (1.0-31.0)
Time to surgery from causative procedure, d		
Mean	—	14 ± 12.0
Median	—	13 (2.0-34.0)
Surgery success	—	9 (100%)

CAD, coronary artery disease; CHF, congestive heart failure; PAD, peripheral arterial disease; PCI, percutaneous coronary intervention; TR, Terumo Radial.

## Discussion

We report several new findings related to RAP presentation, management, and outcomes (Central Illustration). The RAPs were diagnosed relatively late after the index procedure, with only 40% being diagnosed within the first week. However, the first symptoms were reported much earlier than when the diagnosis was confirmed.

Thrombin injection, which has been used to significant effect as a noninvasive management strategy for femoral artery pseudoaneurysms, was used only for one patient in our study. Prior reports, although limited, have also shown infrequent use of thrombin injection.^8^^,^^14^ This is likely due to concern for distal embolization after thrombin injection into RAPs, given their short neck and small size, especially relative to femoral artery pseudoaneurysms. Data on the safety and efficacy of thrombin injection as a treatment modality for RAPs are limited to case reports. For example, Xu et al^12^ reported a short case series of 3 patients with radial artery pseudoaneurysm treated with ultrasound-guided thrombin injection. Two of their 3 patients failed thrombin injection and ultimately went on to require surgical repair.

This study is the first to document the success rate of compression therapy for RAP and predictors of success. Although compression was attempted in almost half the patients, most patients eventually required surgical repair. Failure of compression therapy may in part be related to a lack of a long neck and perhaps a lack of soft tissue surrounding the neck. The fact that a great majority of patients with failed compression were on anticoagulation also likely contributed. Patients treated initially with compression presented earlier and had smaller initial pseudoaneurysm size. Factors associated with the success of compression were an earlier presentation and a smaller pseudoaneurysm size. The former likely suggests a less organized RAP wall being more amenable to compression-induced closure.

A noteworthy finding is that the diagnosis of the RAP was made significantly later than the first symptom onset. This may be related to the fact that, with no symptoms of hand ischemia and less concern for major complications (such as with groin access), patients were often asked to observe and then report a few days later. This finding appears to be an area for education and improvement of care so as to detect the RAP earlier, as that will likely translate into a higher success rate for compression therapy.

Higher BMI and younger age were also associated with a higher likelihood of successful compression. Prior studies on femoral artery pseudoaneurysm have not shown obesity to be protective against failure of compression treatment.^15^ Excess adipose tissue could provide some level of mechanical support during compression. Younger age may also indicate more compressive and less calcified arteries that lend themselves to more effective compression.

The success of compression correlated inversely with the size of the pseudoaneurysm, which is concordant with prior data on compression treatment of femoral artery pseudoaneurysm.^15^ There is a paucity of data about compression strategies in relation to radial artery pseudoaneurysm. There have been case reports of patients on anticoagulation treated successfully with prolonged compression. For example, Molina-Lopez et al^16^ present a case where prolonged manual compression was used along with serial ultrasounds to assess for flow in the pseudoaneurysm. This process took about 3 hours. Most centers are unlikely to compress for this long; however, with radial bands, this is easily achievable. We did not document the length of compression in our study. The benefit of prolonged compression should be weighed against the risk of potential radial artery occlusion, which has previously been associated with prolonged compression.^17^

We also observed that over 60% of the RAPs occurred in women. We do not have the exact percentages of men and women undergoing catheterization procedures during the time studied. However, of the 10,873 coronary angiography procedures performed in the University of Kansas labs between May 2022 and January 2025, only 34.7% of patients were women. Thus, our findings suggest a significantly higher rate of occurrence of RAP in women compared with men. The reasons for this finding are unclear. This disproportionate occurrence of RAP in women may be related to smaller arterial size and possibly to a higher number of RAA attempts, as in the majority of patients, the radial access was obtained without ultrasound guidance.

The rate of surgical intervention in our patients was higher than has typically been seen in the clinical management of femoral artery pseudoaneurysms. Previous studies of patients with femoral artery pseudoaneurysms have shown that the majority of these patients are treated with either local compression or thrombin injection.^18^^,^^19^ The existing literature on this topic is mixed. Burzotta et al^14^ reported that 2 of 12 patients with RAP required surgical intervention, whereas Din et al^8^ reported that 7 of 10 patients with RAP underwent surgical repair. Several factors may contribute to higher use of surgical intervention for RAPs, such as the infrequent use of thrombin injection and noninvolvement of a cardiology team in the care of the RAP on presentation. This occurred more often when the RAP was related to noncoronary procedures, such as arterial lines.

Cardiologists may be more likely to approach a compression-first strategy for RAP management due to their familiarity with radial access and radial compression bands (although this is changing with more radial procedures being performed by vascular surgery teams as well). Finally, our study also includes patients with pseudoaneurysms not related to catheterization, where clinical suspicion for pseudoaneurysm may be lower, resulting in delayed diagnosis, and where surgical repair may be more successful.

Based on our findings and clinical experience, we believe that most patients with RAP should undergo an initial trial of local compression, particularly those presenting early and with a smaller pseudoaneurysm size. Whenever feasible, holding anticoagulation before compression and after the onset of symptoms may improve the likelihood of successful compression. Prevention strategies should include routine consideration of ultrasound-guided radial access, which may reduce the number of puncture attempts, facilitate a clean anterior wall entry, and allow real-time assessment of arterial size. Although further prospective studies are needed, these steps may reduce RAP incidence and improve nonsurgical management success.

Our study has several limitations, including its single-center, retrospective nature and the small sample size, which limited the utility of comparative analysis. However, although the study is small, it is still the largest study on the topic to date and thus provides clinically meaningful information. Another limitation is that since we do not have the total number of procedures performed during the study period, we are unable to determine the incidence of RAP from radial access procedures. The study also did not assess specifics of compression techniques and thus is unable to identify if success rates vary between compression techniques. Larger prospective studies are needed to better understand the incidence, risk factors, and appropriate management strategies for the treatment of access-related RAP.

## Conclusion

Study findings show that RAPs often present several days after the index procedure, although the symptoms occur much earlier. RAPs occur disproportionately in women and those on anticoagulation. Compression is successful in only half the patients. Younger age, earlier presentation, and smaller RAP size are associated with a higher closure success rate.

## Declaration of competing interest

The authors declared no potential conflicts of interest with respect to the research, authorship, and/or publication of this article.
