# Supplementary material for: Clinical Presentation, Management, and Outcomes of Access-Related Radial Artery Pseudoaneurysms: A Single-Center, Retrospective Cohort Study
Source: J Soc Cardiovasc Angiogr Interv. 2025 Dec 9;5(1):104065. doi: 10.1016/j.jscai.2025.104065 (PMC13033806; doi:10.1016/j.jscai.2025.104065)
Supplement: Supplementary Tables 1 and 2 [file mmc1.docx]

Supplement One

**Supplemental Table S1.** Comparing patient characteristics and pseudoaneurysm details for patients who underwent compression vs. those who did not for initial management of radial pseudoaneurysm

|  | **Initial Compression** | **No initial compression** | **p-value** |
| --- | --- | --- | --- |
|  | **(N=19)** | **(N=16)** |  |
| *Patient characteristics* |  |  |  |
| **Age (years) – Mean (SD)** | 65.6 (18.7) | 71.6 (10.5) | 0.2 |
| **Sex** |  |  | 1.0 |
| Male | 7 (36.8%) | 6 (37.5%) |  |
| Female | 12 (63.2%) | 10 (62.5%) |  |
| **Race** |  |  | 1.0 |
| Black or African American | 1 (5.3%) | 1 (6.3%) |  |
| White | 18 (94.7%) | 15 (93.8%) |  |
| **Weight (kg) - Mean (SD)** | 83.6 (32.0) | 80.0 (24.6) | 0.7 |
| **Height (cm) - Mean (SD)** | 167.1 (12.1) | 166.3 (12.0) | 0.8 |
| **BMI (kg/m^2^) – Mean (SD)** | 29.7 (9.9) | 28.7 (8.1) | 0.75 |
| *Comorbidities* |  |  |  |
| **Hypertension** | 15 (78.9%) | 9 (56.3%) | 0.3 |
| **Diabetes** | 3 (15.8%) | 4 (25.0%) | 0.7 |
| **PAD** | 4 (21.1%) | 0 (0%) | 0.1 |
| **CHF** | 9 (47.4%) | 6 (37.5%) | 0.7 |
| **Hyperlipidemia** | 9 (47.4%) | 7 (43.8%) | 1.0 |
| **CAD** | 10 (52.6%) | 7 (43.8%) | 0.7 |
| **Tobacco use** | 9 (47.4%) | 6 (37.5%) | 0.7 |
|  |  |  |  |
| *Pseudoaneurysm details* |  |  |  |
| **Time to ultrasound diagnosis (days) – median [IQR]** | 1.0 [0, 11.5] | 33.5 [21.8, 62.5] | <0.01 |
| **Symptom** |  |  |  |
| Pain | 10 (52.6%) | 6 (37.5%) | 0.5 |
| Swelling | 16 (84.2%) | 15 (93.8%) | 0.6 |
| Numbness | 0 (0%) | 2 (12.5%) | 0.2 |
| Bruising/discoloration | 1 (5.3%) | 1 (6.2%) | 1.0 |
| **Causative procedure** |  |  | 0.7 |
| Heart Catheterization | 15 (78.9%) | 10 (62.5%) |  |
| Arterial Blood Gas | 1 (5.3%) | 2 (12.5%) |  |
| Arterial Line | 2 (10.5%) | 2 (12.5%) |  |
| Other | 1 (5.3%) | 2 (12.5%) |  |
| **Undergone PCI – amongst patients with heart catheterization** | 1 (6.7%) | 4 (40%) | 0.1 |
| **Ultrasound guided arterial access** | 4(26.7%) | 3 (30%) | 0.6 |
| **Initial size – max diameter (cm) - mean (SD)** | 2.0 (1.5) | 2.4 (2.0) | 0.5 |
| **Presence of hematoma** | 3 (15.8%) | 0 (0.0%) | 0.2 |
| **Compression success** | 9 (47.4%) | NA |  |
| **Treatment with intervention** | 9 (47.4%) | 12 (75%) | 0.2 |
| **Intervention type** |  |  | 0.2 |
| Thrombin Injection | 0 (0%) | 1 (8.3%) |  |
| Surgical Repair | 9 (100%) | 11 (91.7%) |  |
| NA | 10 | 4 |  |
| **Time to surgery from symptom onset(days) – Median [IQR]** | 6 [3.0, 14.0] | 65 [16.0, 92.0] | 0.3 |
| **Time to surgery from causative procedure(days) – Median [IQR]** | 13 [4.0, 19.0] | 67 [41.0, 105.0] | <0.01 |
| **Surgery success** | 9 (100%) | 10 (90.9%) |  |
| **Exposure to anticoagulation** | 11 (57.9%) | 10 (62.5%) |  |
| *Abbreviations – CAD (coronary artery disease), CHF (congestive heart failure), PAD (peripheral arterial disease), PCI (percutaneous coronary intervention)* | | |  |

**Supplemental Table S2.** Comparing patient characteristics and pseudoaneurysm details for patients in whom compression was successful vs. not when used for initial management of radial pseudoaneurysm

|  | **Success** | **No success** | **p-value** |
| --- | --- | --- | --- |
|  | **(N=9)** | **(N=10)** |  |
| *Patient characteristics* |  |  |  |
| **Age (years) – mean (SD)** | 61.3 (17.0) | 69.4 (20.3) | 0.3 |
| **Sex** |  |  | 0.1 |
| Male | 5 (55.6%) | 2 (20.0%) |  |
| Female | 4 (44.4%) | 8 (80.0%) |  |
| **Race** |  |  | 1.0 |
| Black or African American | 0 (0%) | 1 (10.0%) |  |
| White | 9 (100%) | 9 (90.0%) |  |
| **Weight (kg) – mean (SD)** | 101.8 (34.4) | 67.2 (19.2) | 0.01 |
| **Height (cm) - Mean (SD)** | 169.0 (9.8) | 165.3 (14.1) | 0.5 |
| **BMI (kg/m^2^) – Mean (SD)** | 35.4 (10.8) | 24.5 (5.4) | 0.01 |
| *Comorbidities* |  |  |  |
| **Hypertension** | 7 (77.8%) | 8 (80.0%) | 1.0 |
| **Diabetes** | 2 (22.2%) | 1 (10.0%) | 0.58 |
| **PAD** | 1 (11.1%) | 3 (30.0%) | 0.58 |
| **CHF** | 5 (55.6%) | 4 (40.0%) | 0.66 |
| **Hyperlipidemia** | 4 (44.4%) | 5 (50.0%) | 1.0 |
| **CAD** | 7 (77.8%) | 3 (30.0%) | 0.07 |
| **Tobacco use** | 6 (66.7%) | 3 (30.0%) | 0.18 |
| *Pseudoaneurysm details* |  |  |  |
| **Time to ultrasound diagnosis (days) – median [IQR]** | 1.00 [0, 1.0] | 6.50 [1.3, 16.8] | 0.09 |
| **Ultrasound diagnosis within 7 days from causative procedure** | 8 (88.9%) | 5 (50%) | 0.14 |
| **Symptom** |  |  |  |
| Pain | 6 (66.7%) | 4 (40.0%) | 0.37 |
| Swelling | 9 (100%) | 7 (70.0%) | 0.21 |
| Bruising/discoloration | 0 (0%) | 1 (10.0%) | 1.0 |
| **Causative procedure** |  |  | 1.0 |
| Heart Catheterization | 8 (88.9%) | 7 (70.0%) |  |
| Arterial Blood Gas | 0 (0%) | 1 (10.0%) |  |
| Arterial Line | 1 (11.1%) | 1 (10.0%) |  |
| Other | 0 (0%) | 1 (10.0%) |  |
| **Undergone PCI – amongst patients with heart catheterization** | 1 (12.5%) | 0 (0%) | 1.0 |
| **Ultrasound guided arterial access** | 2 (25.0%) | 2 (28.6%) | 0.82 |
| **TR band placed (at any time)** | 9 (100%) | 9 (90.0%) | 1.0 |
| **Initial use of TR band (post-LHC)** | 8 (88.9%) | 8 (80.0%) | 1.0 |
| **Initial size – max diameter (cm) - mean (SD)** | 1.63 (1.30) | 2.36 (1.61) | 0.3 |
| **Presence of hematoma** | 2 (22.2%) | 1 (10.0%) | 0.58 |
| **Exposure to anticoagulation** | 4 (44.4%) | 7 (70.0%) | 0.37 |
| **Time to compression from symptom onset (days)** |  |  |  |
| Mean (SD) | 0.8 (2.0) | 4.8 (9.6) |  |
| Median [IQR] | 0 [0, 0] | 0 [0, 2.3] | 0.56 |
| **Time to compression from causative procedure (days)** |  |  |  |
| Mean (SD) | 7.1 (19.5) | 9 (11.5) |  |
| Median [IQR] | 1 [0, 1] | 2.5 [0.3, 16.0] | 0.79 |
| **Treatment with intervention** | NA | 9 (90.0%) |  |
| **Intervention type** |  |  |  |
| Surgical Repair | NA | 9 (100%) |  |
| **Time to surgery from symptom onset (days)** |  |  |  |
| Mean (SD) | NA (NA) | 9.7 (10.0) |  |
| Median [Min, Max] | NA [NA, NA] | 6.0 [1.0, 31.0] |  |
| **Time to surgery from causative procedure (days)** |  |  |  |
| Mean (SD) | NA (NA) | 14 (12.0) |  |
| Median [Min, Max] | NA [NA, NA] | 13 [2.0, 34.0] |  |
| **Surgery success** | NA | 9 (100%) |  |
| *Abbreviations – CAD (coronary artery disease), CHF (congestive heart failure), PAD (peripheral arterial disease), PCI (percutaneous coronary intervention), TR (Terumo Radial)* | | |  |
